# Successful resolution of symmetrical peripheral gangrene after severe acute pancreatitis: a case report

**DOI:** 10.1186/s13256-015-0688-3

**Published:** 2015-09-17

**Authors:** Chen-Yi Liao, Shih-Chung Huang, Cheng-Hui Lin, Chih-Chiang Wang, Mei-Yu Liu, Ren-Jy Ben, Wu-Hsien Kuo, Ching-Chang Lee

**Affiliations:** Department of Internal Medicine, Kaohsiung Armed Forces General Hospital, Kaohsiung, Taiwan

**Keywords:** Low volemic state, Severe acute pancreatitis, Symmetrical peripheral gangrene (SPG)

## Abstract

**Introduction:**

Symmetrical peripheral gangrene is an uncommon but devastating complication in critically ill patients, and it has a high mortality. It is seen in a wide variety of medical conditions, presenting as symmetrical gangrene of two or more extremities without large blood vessel obstruction.

**Case presentation:**

We report a case of a 44-year-old Chinese man who was diagnosed with alcohol-related severe acute pancreatitis and presented with systemic inflammatory response syndrome and intractable vomiting. On the fourth day of admission, the patient developed cyanosis and gangrene of the fingers bilaterally. His cyanosis and gangrene did not resolve despite tapering of the vasopressor treatment. Gradually, his digital gangrene improved after administration of anti-platelet medication and pentoxifylline.

**Conclusions:**

To the best of our knowledge, this is the first case report of symmetrical peripheral gangrene occurring after acute pancreatitis with successful resolution. We highlight the importance of prompt and aggressive fluid resuscitation and consideration of multiple treatment options to prevent a hypovolemic state caused by acute pancreatitis.

## Introduction

Severe acute pancreatitis is a serious condition that can result in both local and systemic complications. Systemic complications can involve the gastrointestinal (GI), cardiovascular, renal, hepatic, and neurological systems, as well as the vascular system. Such complications add to morbidity and mortality, but they have rarely been characterized in symmetrical peripheral gangrene (SPG).

SPG, sometimes termed *purpura fulminans*, is uncommon but not rare in critically ill patients. It is characterized by a distal ischemic change of two or more extremities without large blood vessel occlusion or vasculitis. The latter complication is usually observed in the setting of disseminated intravascular coagulation (DIC), sepsis, low cardiac output state, collagen vascular disease, anti-phospholipid syndrome, and drugs such as ergot, vasopressin, and noradrenaline [1–3]. It is rarely associated with acute pancreatitis. In this report, we describe a case of a man who developed SPG as a complication of severe acute pancreatitis.

## Case presentation

A 44-year-old Chinese man, who was an occasional smoker and social drinker, presented with complaints of acute pain in his upper abdomen and vomiting of particulate material resembling coffee grounds during the 24h before admission. His pain had developed 8–10h after consumption of Kaoliang liquor (3000ml of homemade liquor consisting of approximately 58% alcohol) over a period of 3 days while at home. Initially, he presented with epigastric pain, nausea, vomiting, and watery diarrhea. Because of aggravation of epigastric pain with subsequent vomiting of coffee ground–like material and passage of tarry stools, he was admitted to the emergency room by ambulance. His pain was dull, aching, and became aggravated upon eating. He had intractable vomiting approximately 30 times and subsequently vomited coffee ground–like material on the day of admission. His past medical history included two episodes of alcohol-related acute pancreatitis without mention of diabetes mellitus, claudication, cardiovascular disease, or illicit drug abuse. The patient denied steatorrhea, maldigestion, or recent body weight loss, which excluded possible chronic pancreatitis.

His initial vital signs revealed blood pressure of 74/27mmHg, heart rate of 152 beats/min, and body temperature of 38.7 °C. His clinical picture was compatible with systemic inflammatory response syndrome (SIRS) with shock status. His physical examination revealed that his abdomen was mildly distended with tenderness over the epigastric area. Chest, extremity, and other systemic examinations were unremarkable. Routine laboratory investigations revealed a hemoglobin level of 11.6g/dl, white blood cell count of 14,000/μl, amylase of 680U/L, lipase of 1125U/L, albumin of 2.0g/dl, total bilirubin of 0.61mg/dl, alkaline phosphatase of 110U/L, γ-glutamyl transferase of 70U/L, C-reactive protein of 24.49mg/dl, lactate dehydrogenase of 362U/L, and creatine phosphokinase of 489U/L. He had impaired renal function with blood urea nitrogen of 28mg/dl and creatinine of 2.3mg/dl. Impaired liver function with aspartate transaminase of 250U/L and alanine transaminase of 47U/L were also noted. He had high anion gap metabolic acidosis characterized by a lactic acid level of 5.75mmol/L, arterial blood pH of 7.058, bicarbonate level of 5.1mmol/L, anion gap of 32.1, and ethanol of 116mg/dl. His laboratory test result for serum ketone bodies was negative. His coagulation profile was consistent with SIRS with shock status and elevated D-dimer levels. His initial central venous pressure was recorded as 4mmHg. A diagnostic screen for sepsis showed unremarkable blood culture results (Table 1). His Ranson’s criteria scores were 3 on admission and 2 after 48h. Ultrasonography and computed tomography (CT) of the abdomen and abdomen revealed a picture compatible with acute pancreatitis, CT severity index grade E.

**Table 1:** Survey for symmetrical peripheral gangrene

Laboratory test (normal values)	Laboratory value
HBsAg	Nonreactive
HCV antibody	Nonreactive
Cryoglobulin	Negative
ESR, mm/h (0–15)	69mm/h
RPR/VDRL test	Negative
Cold agglutinin antibodies (<1:16×(−); 1:16X(+))	<1:16
C3, mg/dl (86–160)	143
C4, mg/dl (17–45)	30
Protein C, % (70–140%)	78.7
Anti-thrombin III, % (75–125%)	73.4
c-ANCA, IU/ml (<2)	0.1
ANA (<1:20)	<1:20
Anti-DNA, <Day 12 after admission10(−); 10–15(+/−); >15(+)	0.1(−)
Anti-cardiolipin IgM, MPL U/ml (<10)	0.1
Anti-cardiolipin IgG, IU/ml (<10)	0.2
DIC profile	
Platelets (150,000–400,000/μl)	170,000/μl
PT, s (8.0–12.0)	12.5
INR	1.27
aPTT, s (24.3–32.7)	33.1
D-dimer, ng/ml (<500)	4441.30
Fibrinogen, mg/dl (150–350)	559.5
CEA, ng/ml (0–5)	3.77
AFP, ng/ml (1.09–8.04)	3.20
CA 19-9, U/ml (0–37)	6.52
PSA, ng/ml (0–4)	0.622

AFP, α-fetoprotein; ANA, anti-nuclear antibodies; c-ANCA, cytoplasmic anti-neutrophil cytoplasmic antibodies; aPTT, activated partial thromboplastin time; C3, complement component 3; C4, complement component 4; CA 19-9, carbohydrate antigen 19-9; c-ANCA, cytoplasmic anti-neutrophil cytoplasmic antibodies; CEA, carcinoembryonic antigen; DIC, Disseminated intravascular coagulation; ESR, erythrocyte sedimentation rate; HBsAg, hepatitis B surface antigen; HCV, hepatitis C virus; Ig, immunoglobulin; INR, international normalized ratio; PSA, prostate-specific antigen; PT, prothrombin time; RPR/VDRL, rapid plasma regain/venereal disease research laboratory

According to the revised Atlanta classification of pancreatitis, the patient was diagnosed with severe acute pancreatitis due to persistent organ failure (profound shock lasting for more than 48h and acute kidney injury with oliguria status after emergent hemodialysis).

**Fig. 1:**
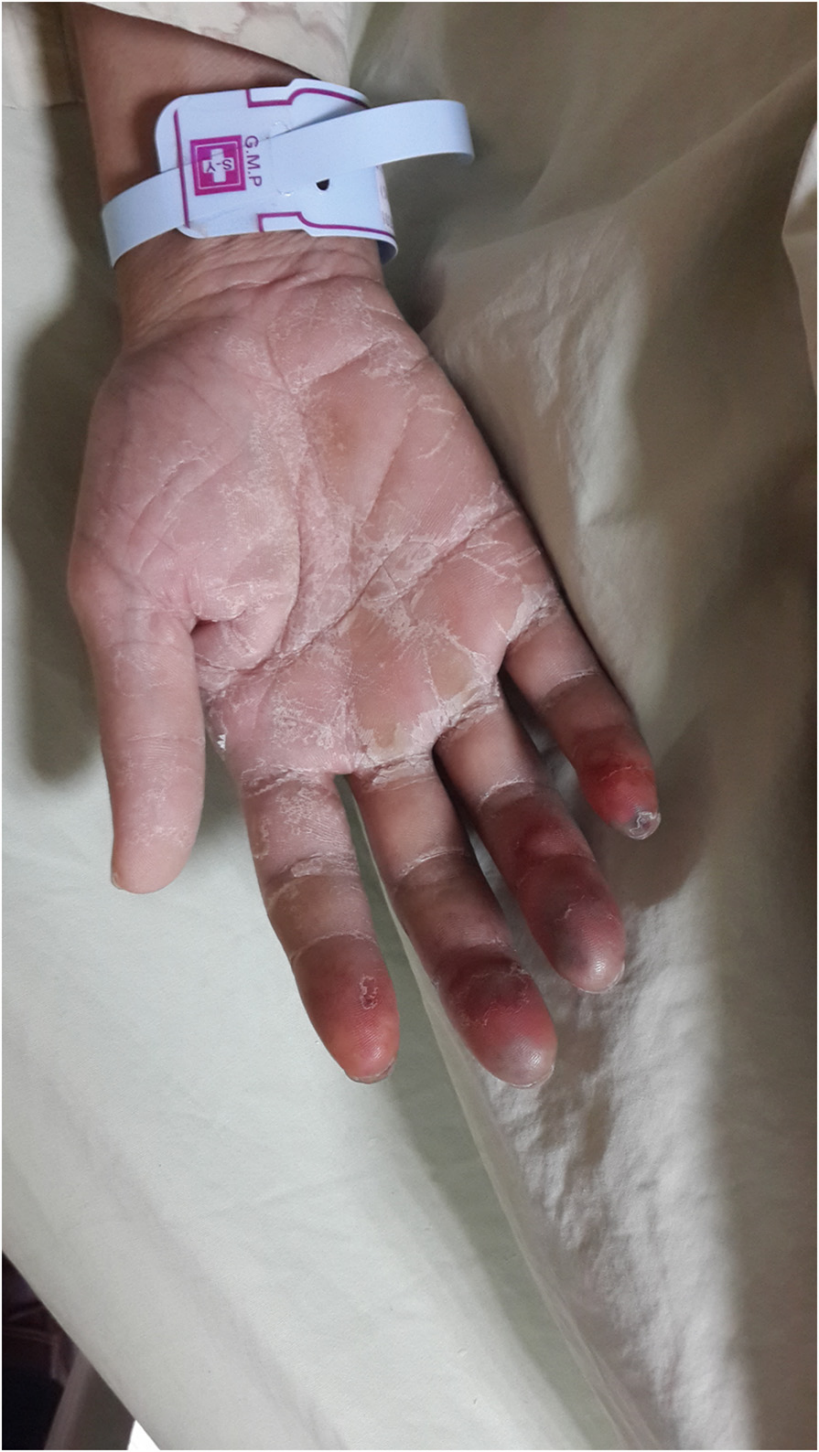
Acrocyanosis on day 9 after admission

He was treated promptly and aggressively with intravenous fluids with normal saline. His intravenous fluids were given as a 20ml/kg bolus, followed by a maintenance dose of 3ml/kg/h. He was also prescribed analgesic for pain relief. Because the patient had profound shock and a poor response to fluid resuscitation, a vasopressor was administered with intravenous norepinephrine at an infusion rate of 4μg/min for 2 days through the central venous line. This was discontinued after his blood pressure was restored. Upper GI panendoscopy was conducted because of vomitus of coffee ground material, which revealed a Mallory-Weiss tear and acute gastric ulcer with a history of recent hemorrhage. An antibiotic (ceftriaxone) and a proton pump inhibitor (omeprazole) were administered. Continuous renal replacement therapy was initiated for refractory acidosis and contrast media removal. The patient responded well to aggressive, conservative management. On the fourth day after hospitalization, a bilateral bluish discoloration of the fingers was noted. A gradual painful sensation developed 5 days after an acrocyanotic lesion was found (Fig. 1).

**Fig. 2:**
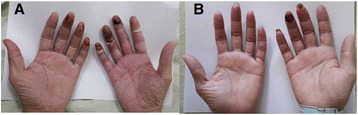
**a** Day 12 after admission: gangrenous change over the fingers on day 9 after acrocynosis noted. **b** Day 26 after admission: resolution of symmetrical peripheral gangrene on day 22 after acrocyanosis noted.

Dry gangrene and a limited range of motion of the fingers developed 9 days after the lesion was noted. Peripheral pulses were palpable in both the upper and lower extremities. The results of laboratory tests for SPG, comprising infection, malignancy, and the autoimmune system, were unremarkable (Table 1). Transesophageal echocardiography, carotid Doppler ultrasound, and sonography of the upper extremity vessels also were unremarkable. The patient was administered two doses of oral aspirin 75mg/day and two doses of oral pentoxifylline 400mg/day for 20 days. Because of upper GI tract bleeding noted on admission, heparin was not considered. The gangrenous lesion was kept warm and was not touched, as much as possible. The patient’s condition improved steadily over the next 7 days. His pain decreased, and his range of motion improved. Gradual desquamation of his finger skin occurred, with shedding of gangrenous scabs from the tips of his fingers and complete resolution (Fig. 2a, b). The patient was discharged from the hospital stay 26 days later. The patient responded well to the treatment and returned to his normal daily activities during outpatient follow-up.

## Discussion

SPG is a relatively rare phenomenon characterized by symmetrical distal ischemic damage that leads to gangrene of two or more sites in the absence of large blood vessel obstruction, where vasoconstriction rather than thrombosis is implicated as the underlying pathophysiology [1, 4]. The gangrenous lesions initially appear in the form of acrocyanotic and dusky discolorations of the skin starting from the distal extremity within 24–48h and resemble lesions associated with erythematous cold extremity exposure. Gangrene most commonly occurs in the distal extremities, such as the fingers, toes, tips of the nose, and ear lobules, whereas the lips or genitalia may be affected in severe cases [5]. Peripheral pulses are usually palpable as a result of sparing of the large vessels. Up to 85% of patients with SPG attributed to DIC and other proposed mechanisms, such as the Shwartzman reaction, bacterial endotoxin release, low flow states, and spastic process of vessels [2, 6–8]. The mortality rate ranges from 30% to 50% of surviving patients, in whom it is common for at least one limb to be amputated [2, 4, 9]. A wide array of infective and non-infective etiological factors have been linked with the development of SPG, such as bacterial and viral infections (e.g., *Clostridium perfringens* and *Neisseria meningitides*), sepsis, shock status, cardiovascular problems, and treatment with vasoactive agents, such as vasopressin, dopamine, and noradrenaline [3].

We conducted a review of the English-language medical literature, which revealed rare occasions of patients experiencing SPG with presentation in only the upper limb precipitated by acute pancreatitis [10, 11]. Blue toe syndrome complicated by acute pancreatitis or arteriopancreatic syndrome may be identical to SPG at different stages of the clinical course.

Acute pancreatitis usually presents with low volemic state, hypercoagulation, and hemoconcentration, which can contribute to low flow and vascular disturbance. This not only leads to local pancreatic microcirculatory impairment but also involves distant microvascular and macrovascular circulation. It has varied presentations, ranging from isolated intravascular thrombosis due to DIC, which may result in vascular occlusion and further progression to SPG.

In our patient, we collected laboratory data that did not resemble DIC. Though high-dose vasopressor treatment related to SPG is well described in the literature [3], we administered norepinephrine to our patient at a suboptimal dose and only for a brief period (2 days) that did no correlate with the timeline of SPG, which developed 2 days after discontinuation of the vasopressor. Thus, we believe that acute pancreatitis in our patient was highly associated with the occurrence of SPG. In patients with acute pancreatitis complicated by SPG, fluid resuscitation should be administered first, instead of vasopressor treatment, except for cases where there is refractory shock status. Prompt fluid resuscitation, empirical antibiotics, and anti-coagulant therapy may improve the flow state and control the underlying infection, which will further alleviate the DIC episode.

## Conclusions

We report this case to highlight the importance of early identification and treatment of pre-gangrenous changes in limbs to avoid amputation. This is the first time, to the best of our knowledge, that imaging has captured the clinical sequence of events from onset of gangrene to localization of a typically disseminating phenomenon. During the regenerative process, meticulous care of the affected areas is required to prevent infections and ensure a favorable outcome.

To the best of our knowledge, this is the first case report of SPG after acute pancreatitis with successful resolution. We highlight the importance of early, aggressive fluid resuscitation and multiple treatment options to prevent a hypovolemic state caused by acute pancreatitis.

## Consent

Written informed consent was obtained from the patient for publication of this case report and accompanying images. A copy of written consent is available for review by the Editor-in-Chief of this journal.

## Abbreviations

AFP: α-fetoprotein
ANA: anti-nuclear antibodies
c-ANCA: cytoplasmic anti-neutrophil cytoplasmic antibodies
aPTT: activated partial thromboplastin time
C3: complement component 3
C4: complement component 4
CA 19-9: carbohydrate antigen 19-9
CEA: carcinoembryonic antigen
DIC: Disseminated intravascular coagulation
ESR: erythrocyte sedimentation rate
GI: gastrointestinal
HBsAg: hepatitis B surface antigen
HCV: hepatitis C virus
Ig: immunoglobulin
INR: international normalized ratio
PSA: prostate-specific antigen
PT: prothrombin time
RPR/VDRL: rapid plasma regain/venereal disease research laboratory
SIRS: Systemic inflammatory response syndrome
SPG: Symmetrical peripheral gangrene
